# Orthodontic Curricula in Undergraduate Dental Education—A Scoping Review

**DOI:** 10.3390/ijerph20064914

**Published:** 2023-03-10

**Authors:** Shailaja Raghavan, Elham Abu Alhaija, Kamran Ali

**Affiliations:** College of Dental Medicine, QU Health, Qatar University, Doha P.O. Box 2713, Qatar

**Keywords:** orthodontic curriculum, learning outcomes, assessments, competency

## Abstract

Objective: To investigate commonalities and variations in the learning outcomes, curriculum content, assessment methods, and competencies in undergraduate orthodontic curricula globally. Methods: This scoping review followed the updated methodological guidance proposed by the Joanna Briggs Institute and reported in accordance with the Preferred Reporting Items for Systematic reviews and Meta-Analyses extension for Scoping Reviews (PRISMA-ScR). A search on electronic databases PubMed, Scopus, and Embase was conducted for the last 25 years. Google Scholar was used to identify eligible unpublished and grey literature. Results: The total number of reports identified was 231. After removal of 62 duplicates, 169 reports were included in the title and abstract screening. Finally, 17 studies were included in the review, which included 13 cross-sectional surveys, three expert panel proceedings, and one discussion paper. Marked variations were reported in undergraduate orthodontic curricula and competency assessments at the level of individual countries, regionally as well as globally. The challenges of imparting competency in orthodontic treatment during undergraduate dental education are also acknowledged. Conclusion: Lack of consistency in undergraduate orthodontic education was evidenced by several Delphi studies aiming to develop a consensus on orthodontic teaching in undergraduate programs. A common message emanating from the available studies on undergraduate orthodontic education seems to emphasize a focus on assessment and diagnosis of the orthodontic treatment needs of patients and a basic understanding of contemporary treatment options to facilitate patient referral.

## 1. Introduction

Orthodontics is a recognized clinical dental specialty and undergraduate dental programs worldwide aim to equip undergraduate students with the core knowledge and skills in orthodontics to be able to provide safe dental care to patients. However, there appear to be significant variations in the scope of orthodontics in undergraduate dental education not only globally but also amongst universities in the same country. These variations may lead to marked differences in curriculum content, teaching methods, and competency assessments [1]. Consequently, dental graduates from different dental schools may demonstrate considerable disparities in the skills to assess, manage, and refer orthodontic patients in general dental practice settings.

General dental practitioners (GDPs) act as gatekeepers for provision of specialist dental services since they are the main source of referral to dental specialists [2]. A study conducted in the United States of America (USA) reported that up to 72% of the general dentists surveyed were performing some form of orthodontics and 38% of those cases involved clear aligners [3]. Therefore, the recognition, timely referral, and optimal management of orthodontic patients is heavily dependent on GDPs. Amongst other factors, undergraduate dental education in orthodontics has a huge influence on the approach taken by GDPs to deal with orthodontic patients. Achieving a broad consensus on orthodontic curricula is crucial for optimal and safe patient care by GDPs.

Regulators of dental education in different regions of the world have outlined the learning outcomes (LOs) for undergraduate orthodontic education. The Association for Dental Education in Europe (ADEE) expects graduating European dentists to be competent in diagnosing orthodontic treatment needs and applying contemporary treatment techniques [4]. The Commission on Dental Accreditation (CODA) on standards of malocclusion and space management formed by the American Dental Association (ADA) requires pre-doctoral graduates to provide comprehensive care experiences to patients commensurate with competence in all components of general dentistry practice [5]. The generic description of LOs by ADEE and CODA leave considerable room for interpretation, which may lead to variations in which academic programs develop and deliver their undergraduate orthodontic curricula and assessments.

There is limited published literature on the content, structure, and delivery of orthodontic curricula by dental schools worldwide, which makes it difficult to evaluate variations among individual programs. Evidence from dental schools in the USA suggests that the knowledge and clinical experience of students tend to vary by program, and presumably, so do competencies and outcomes assessment [6]. The General Dental Council (GDC) in the United Kingdom (UK) appears to provide a more focused description of the Los for undergraduate orthodontic curriculum [7]. In the UK, dental graduates are expected to be competent in assessing orthodontic treatment needs, providing emergency orthodontic treatment, referring patients to orthodontic specialists, and explaining contemporary orthodontic treatment options to patients. In spite of clearly defined undergraduate orthodontic LOs by the GDC, studies on UK dental students have reported a lack of clinical experience in assessing patients, applying the index of orthodontic treatment needs (IOTN), and making referrals [8].

Given the apparent lack of consistency in undergraduate orthodontic curricula, a fundamental question to inform the refinement of orthodontic curricula is: “What knowledge and skills must a general dentist have to properly manage their patients with malocclusion and/or skeletal problems?” [9,10]. Opposing views on the scope of undergraduate orthodontic education are reported in the literature, ranging from those who favor restricting undergraduate orthodontic teaching to the basics [11] to those who support more comprehensive teaching and training [12].

The present scoping review was aimed at mapping the research related to undergraduate (pre-doctoral) orthodontic curricula in order to determine the commonalties and variations in undergraduate orthodontic education and identify any gaps in knowledge.

## 2. Methods

### 2.1. Study Protocol and Registration

This scoping review adopted the updated methodological guidance proposed by the Joanna Briggs Institute (JBI) and reported in accordance with the Preferred Reporting Items for Systematic reviews and Meta-Analyses extension for Scoping Reviews (PRISMA-ScR) [13,14,15]. The protocol was registered in Open Science Framework [16].

### 2.2. Research Question

What is known from the literature regarding the learning outcomes, curriculum content, competencies, and assessments for undergraduate curricula in Orthodontics?

A population-concept-context (PCC) framework was used to answer the research question as explained below:

Population: Orthodontic educators and specialists, undergraduate dental students, and newly qualified dental graduates.

Concept: Orthodontic curriculum, learning outcomes, and assessment methods.

Context: Knowledge and competencies in orthodontics in undergraduate dental programs.

### 2.3. Eligibility Criteria

The eligibility criteria were based on the PCC strategy and are summarized below:

#### 2.3.1. Inclusion Criteria

1. Peer-reviewed journal papers on orthodontic curriculum, learning outcomes, and assessments in undergraduate orthodontic curricula.
2. Published from 1 January 1998 to 31 December 2022.
3. Published in English.

#### 2.3.2. Exclusion Criteria

1. Studies on postgraduate education in orthodontics.
2. Studies published in languages other than English.

### 2.4. Information Sources

A comprehensive electronic database search was conducted up to 31 December 2022 and limited to the last 25 years (1998–2022). Literature from relevant databases such as PubMed, Scopus, and Embase were included in the review. Google Scholar was used to identify eligible unpublished and grey literature. Manual searching was performed from the reference lists of included articles.

### 2.5. Search Strategy

The search strategy comprised a combination of Medical Subject Headings (MeSH) and keywords for PubMed and index terms pertaining to the other databases. The detailed search string was (clinical competence) AND (orthodontics) AND (education*) AND (teaching).

### 2.6. Selection of Sources of Evidence

All of the identified articles were imported into a reference management software (desktop version of EndNote^®^ version X9; Clarivate Analytics, London, UK). After removal of duplicate articles, two reviewers (S.R and E.A.A.) independently screened the articles based on their titles and abstracts using Rayyan Systematic Review Screening Software [17]. The studies with abstracts fitting the eligibility criteria were selected for full text reading for the final selection. Any disagreements were resolved by consensus.

### 2.7. Data Charting Process

Data charting was performed by two reviewers (S.R. and E.A.A.) independently using a data charting sheet to capture the essential data items [13].

### 2.8. Data Items

The data items included the author’s name, year of publication, title, study design, setting, objectives, sample size, population, data collection tool, and conclusion.

### 2.9. Ethics Approval

Ethics approval was not applicable to this scoping review as no new data was generated for this study.

## 3. Results

### 3.1. Study Selection

**Figure 1:**
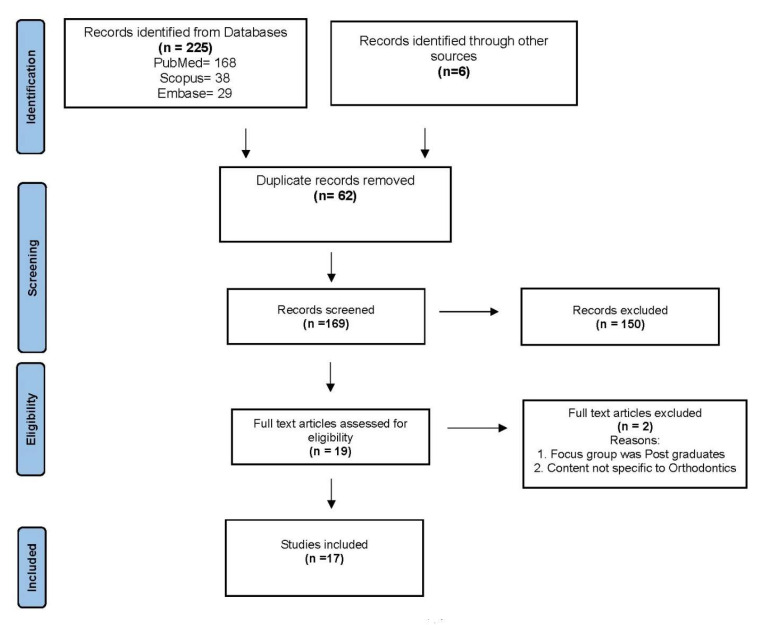
PRISMA flow diagram for scoping review.

The results of the literature search and study selection are shown in Figure 1. The total number of reports identified was 231 (225 reports from databases and 6 reports from grey literature). After removal of 62 duplicates, 169 reports were included in the title and abstract screening. Of these, 147 reports from databases and 3 reports from grey literature were excluded, and only 19 reports (13 from databases and 3 from grey literature) were considered for full text screening. The full texts of potentially eligible articles were assessed for inclusion by the two reviewers, which resulted in the exclusion of two studies [18,19]. Finally, 17 articles [1,6,17,18,19,20,21,22,23,24,25,26,27,28,29,30,31] were included for qualitative analysis.

### 3.2. Study Characteristics

This scoping review included 13 cross-sectional surveys, 3 expert panel proceedings, and 1 discussion paper. Of these, seven of the included studies investigated undergraduate orthodontic learning outcomes and course content [1,6,20,21,22,23,24]; eight studies investigated competency of undergraduate dental students [18,25,27,28,29,30,31,32]; three studies investigated assessment tools as primary outcome [33,34,35]; one study investigated LOs as primary outcome and assessment tools as an additional outcome [1]; and one study evaluated learning outcomes and course content in addition to competency of dental students [20]. The main findings of the studies included in this review are discussed under three themes below.

### 3.3. Theme I: Undergraduate Orthodontic Curricula: Learning Outcomes and Course Content

Article 1: The current state of predoctoral orthodontic education in the United States (Kwo et al., 2011) [1].

This study assessed predoctoral orthodontic education across dental schools in the United States. Twenty-nine program directors (53%) completed an online survey. The outcome of this survey showed that the number of curriculum hours devoted to teaching orthodontics during the predoctoral years varied greatly between schools. Additionally, variations in curriculum content in predoctoral orthodontic programs were observed. Approximately 48% of the programs required students to treat orthodontic patients (56% fixed appliances, 41% functional, and 51% clear aligners). Two thirds of the participating dental schools considered the current time allocated for predoctoral orthodontic clinical education at their institutions to be adequate.

Article 2: Expert consensus on growth and development curricula for predoctoral and advanced education orthodontic programs (Ferrer et al., 2019) [21].

This study aimed to obtain expert consensus on growth and development topics in predoctoral education programs in orthodontics and determine the level of cognition on the subtopics necessary to demonstrate learner competence. The results of the consensus showed that the desired level of cognition for predoctoral students was “understand.”

Article 3: Expert consensus on didactic clinical skills development for orthodontic curricula (Ferrer et al., 2021) [6].

This study aimed to determine the curriculum content and competency in predoctoral orthodontic programs. A modified Delphi approach was used and a focus group, composed of five full-time faculty members from three orthodontic programs, was formed to develop a predoctoral orthodontic curriculum. Seven key topic areas in undergraduate orthodontic curricula were identified, including production of diagnostic records; dentition analyses; classification, etiology, and epidemiology of malocclusion; relationship of morphology to malocclusion; management of malocclusion; tooth movement; and appliances. The group deliberated to develop a consensus on the relevance of topics in undergraduate orthodontic curricula. Their results identified that all topics proposed by the focus group were necessary, except for appliances. They concluded that to be deemed competent, new graduates must demonstrate knowledge in the cognitive domain of “understand,” including the principles of application of orthodontic appliances.

Article 4: Orthodontic teaching practice and undergraduate knowledge in British dental schools (Rock et al., 2002) [20].

This study was conducted to investigate orthodontic teaching practices in the undergraduate curricula at British dental schools and evaluate the abilities of undergraduate students according to the requirements of the GDC, UK. Data were collected by means of a questionnaire sent to each dental school in 1998 and compared with similar data from 1994. The orthodontic knowledge and treatment planning ability of students were assessed by a multiple-choice examination paper completed by a random 10% sample of students from each dental school. The results showed that the teaching of fixed appliances had increased considerably between 1994 and 1998. Students scored well on questions that tested basic knowledge but performed poorly on questions related to the application of knowledge. They concluded that undergraduate orthodontic training should concentrate on diagnosis and recognition of problems rather than on providing limited exposure to treatment techniques.

Article 5: A survey of undergraduate orthodontic education in 23 European countries (Adamidis et al., 2000) [22].

This study explored the teaching contents of the undergraduate orthodontic curricula in European countries and whether or not these countries set a formal undergraduate examination in orthodontics. A questionnaire was mailed to all members of the EURO-QUAL BIOMED II project (23 countries). The results showed that the time allocated to orthodontic teaching, including theory, clinical practice, laboratory work, diagnosis, and treatment planning, varied widely. In general, clinical practice and theory were allocated higher number of teaching hours, whilst diagnosis, laboratory work, and treatment planning were reported to receive relatively less time. Removable appliances were reported to be taught in 22 of the 23 countries, functional appliances in 21 countries, and fixed appliances in 17 countries. An undergraduate examination in orthodontics was reported by 20 countries.

Article 6: Developing faculty consensus for undergraduate orthodontic curriculum (Bashir et al., 2017) [23].

This study employed a qualitative methodology to develop faculty consensus of orthodontic learning outcomes associated with the knowledge and skills expected from undergraduate students. Learning outcomes related to skills were formulated in the form of a questionnaire and sent to study participants. The faculty rated the skills in treatment on a five-point Likert scale. The orthodontic faculty agreed that undergraduate students must have skills in history taking, oral examination, radiographs, and removable appliances.

Article 7: Orthodontic curriculum in Saudi Arabia: Faculty members’ perception of clinical learning outcomes (Al Gunaid et al., 2021) [24].

This study aimed to assess the perceptions of orthodontic staff members about clinical LOs of the undergraduate orthodontic curriculum with a focus on dental schools in Saudi Arabia. Twenty-three LOs were formulated, all of which were associated with skills required in the undergraduate orthodontics course. Orthodontic staff members were invited to provide their opinion regarding the curriculum using a five-point Likert scale. Sixty-one teaching staff members participated in this study. The highest level of agreement among the participants was related to conducting systematic orthodontic intraoral and extraoral examinations (100%), followed by explaining causes for space loss (98.3%). Approximately 67.1% of the academics did not expect dental students to undertake fixed appliance therapy.

### 3.4. Theme II: Competency of Undergraduate Dental Students

Article 1: Preparedness of undergraduate dental students in the United Kingdom: a national study (Ali et al., 2017) [25].

This study evaluated the self-perceived preparedness of final year dental undergraduate students in the United Kingdom. The participants were required to rate their self-perceived preparedness on a validated 50-item Dental Undergraduates Preparedness Assessment Scale [26], which was administered online. The key finding relevant to the present study was that participants felt underprepared to assess the orthodontic treatment needs of patients.

Article 2: The undergraduate preparation of dentists: Confidence levels of final year dental students at the school of dentistry in Cardiff (Gilmour, 2016) [27].

This study investigated the self-reported confidence and preparedness of final year undergraduate students in undertaking a range of clinical procedures. A questionnaire was distributed to final year dental students at Cardiff University, UK, six months prior to graduation. The results showed that 80% of dental students felt unprepared for clinical work and participants’ confidence scores were lower for complex procedures that were least practiced. The lowest confidence was reported on design/fit/adjustment of orthodontic appliances.

Article 3: Does reflective learning with feedback improve dental students’ self-perceived competence in clinical preparedness? (Ihm, 2016) [28].

This study aimed to explore whether reflective learning with feedback enabled dental students to assess their self-perceived levels of preparedness. Over 16 weeks, all third- and fourth-year students at a dental school in the Republic of Korea took part in clinical rotations that incorporated reflective learning and feedback. Following this educational intervention, the participants self-reported on clinical competence. The results showed that compared to other clinical dental disciplines, the participants were least confident in providing orthodontic care.

Article 4: Dental students’ experiences of treating orthodontic emergencies—a qualitative assessment of student reflections (Jones, 2015) [29].

The outcome of interest for this study was to evaluate dental students’ experiences of treating orthodontic emergencies at a teaching institution. This study was designed as a single-center evaluation of teaching based at a UK university’s orthodontic department. The participants were fourth-year dental students who treated orthodontic emergency patients under clinical supervision as part of the undergraduate curriculum. Student logbook entries for one academic year detailing the types of emergencies treated were analyzed. The results showed that the majority of dental students (69%) were confident in managing orthodontic emergencies. The study concluded that theoretical knowledge in orthodontics supplemented with exposure to a range of clinical problems within a supported learning environment made students feel more confident.

Article 5: Undergraduate training as preparation for vocational training in England: a survey of vocational dental practitioners’ and their trainers’ views (Patel et al., 2006) [30].

This study aimed to identify areas of weakness in dental undergraduate education that could influence the future training needs of vocational trainees (VTs) using structured postal questionnaires. The results showed that a large proportion of VTs and their trainers perceived undergraduate training in orthodontics to be inadequate and did not provide adequate skills to the students.

Article 6: Orthodontic competency in predoctoral education in American dental schools (Oesterle and Belanger, 1998) [31].

The aim of the study was to investigate the impact of a competency-based educational approach in predoctoral orthodontic education in the USA. A questionnaire was mailed to orthodontic departments at 53 dental schools. The results showed that only 61% of schools assessed clinical competency in orthodontics. However, these assessments were based on formative clinical evaluations in most participating dental schools. Only 24% of schools used a summative competency examination consisting of a written examination, an oral examination, or both. Some schools also evaluated student performance on specific procedures, such as fitting bands, while others evaluated patient diagnoses and treatment planning. Approximately 16% of those responding did not evaluate orthodontic competency. The study recommended that predoctoral orthodontic education should focus more on skills to diagnose and “manage” orthodontic patients and less on providing active treatment.

Article 7: Competence profiles in undergraduate dental education: a comparison between theory and reality (Koole et al., 2017) [32].

This study aimed to investigate whether a competence profile as proposed by academic and clinical experts was able to represent clinical reality. A questionnaire was developed including questions about gender, age, and perception about required competences. A total of 312 questionnaires were completed. All competences in the European competence profile were rated between 7.2 and 9.4 on a 10-point scale. The results showed that restorative dentistry, prosthodontics, endodontics, pediatric dentistry, and periodontology were reported to be the most important domains in undergraduate education. In regard to orthodontics, assessment of patients’ treatment needs was the key skill required during undergraduate education.

Article 8: Orthodontic teaching practice and undergraduate knowledge in British dental schools (Rock et al., 2002) [20].

The orthodontic knowledge and treatment planning ability of students were assessed by a multiple-choice examination paper completed by a random 10% sample of students from each dental school. Students scored well on questions that tested basic knowledge, but scores were low for questions testing the application of knowledge to clinical problem-solving in orthodontics. The findings of this study suggested that undergraduate orthodontic training should focus on learning to diagnose orthodontic problems rather than providing limited exposure to treatment techniques.

### 3.5. Theme III: Assessment Methods in Undergraduate Orthodontic Curricula

Article 1: The current state of predoctoral orthodontic education in the United States (Kwo et al., 2011) [1].

This study assessed predoctoral orthodontic education in the USA. Marked variations in methods of assessment in predoctoral orthodontic programs were found. All schools reported using written examinations for assessment. Laboratory and clinical examinations were reportedly used to assess undergraduate students in 74% and 65% of schools, respectively. Only 27% of schools reported the use of objective structured clinical examinations (OSCE) in orthodontics. However, limited information was provided regarding the value of individual assessment methods.

Article 2: A Process for Developing Assessments and Instruction in Competency-Based Dental Education (Lipp, 2010) [33].

This paper described the process of assessments in competency-based dental education using an orthodontic module as an example. Although this paper was related to strategic planning of competency-based assessments in general, use of orthodontic topic areas as examples suggested that it may also be effective for orthodontic assessments.

Article 3: Test-Enhanced Learning in Competence-Based Pre-doctoral Orthodontics: A Four-Year Study (Freda, 2016) [34].

This study evaluated the impact of a test-enhanced instructional approach on competence in diagnosis and management of dental and skeletal malocclusion among third year dental students at the New York University College of Dentistry. The results showed improved performance of students in diagnosis and treatment planning of malocclusion using the test-enhanced instructional method despite no significant differences in the pass rates of the participants.

Article 4: Recognition of malocclusion: An education outcomes assessment (Brightman, 1999) [35].

This study aimed to assess the outcome of undergraduate orthodontic education in the school of dentistry at Case Western Reserve University in Cleveland, OH, USA. A test tool was employed to ascertain the abilities of predoctoral students in diagnosing malocclusion using clinical records of seven children. In addition, students’ knowledge was tested using orthodontic questions chosen from national board examinations. The results showed only slight differences among fourth- and first-year undergraduate students, demonstrating that existing assessments methods were not appropriate to measure dental students’ diagnostic and referral skills. This study underscored the need for increased clinical training to develop competence in the diagnosis and referral of malocclusions. Additionally, a need for appropriate assessments was identified.

## 4. Discussion

There is limited research on the structure and delivery of orthodontic curricula in undergraduate dental education. The results of this study highlighted marked variations in the LOs, curriculum content, competencies, and assessments of undergraduate orthodontic curricula not only at global and regional levels but also in individual countries with institutions working under a single regulator. These findings underscore the need to revisit undergraduate orthodontic curricula to improve consistency and public confidence regarding the remit and competence of GDPs in providing orthodontic assessment and management. Dental institutions and regulators need further work to achieve constructive alignment in the orthodontic curricula and intended outcomes, the teaching methods used, and the methods used to assess the competence of undergraduate dental students [36]. Such an approach can help to achieve parity in the scope of orthodontic services provided by GDPs in primary care settings.

The findings of this study corroborate those of previous studies on predoctoral students in the USA that have identified marked variations in orthodontic curricula, teaching methods, clinical exposure, competencies, and assessments of students in dental schools across the USA [1,31]. Similar findings were reported in a previous study involving 23 European dental schools [22]. The orthodontic competence for European dentists outlined by the ADEE requires dental graduates to be able to diagnose orthodontic treatment needs and be “competent with contemporary treatment techniques” [4]. Orthodontics is a specialized field, and it may be unrealistic to achieve competence in contemporary orthodontic treatment needs during undergraduate dental education. It may be argued that specialized postgraduate training in orthodontics is also aimed at achieving competence in contemporary orthodontic treatment techniques and expecting undergraduates to achieve the same appears to blur the boundaries between the remit of GDPs and specialist orthodontists. It may be difficult for dental institutions to achieve the orthodontic LOs defined by the ADEE in the limited time available for clinical training in undergraduate dental programs. It is suggested that the ADEE revisit the LOs related to orthodontics and elaborate further on competence in contemporary treatment techniques to enhance clarity for education providers.

The orthodontics LOs for undergraduate dental students defined by the GDC appear to be focused and easy to interpret [7]. However, previous studies on UK dental students and new graduates have shown that a significant proportion of participants were not confident in assessing the orthodontic treatment needs of patients [25] and managing orthodontic appliances [27]. In recent years, similar findings were reported on dental graduates from other countries [37]. Al Gunaid et al. reported consensus among orthodontic staff that orthodontic intraoral and extraoral examinations be included in the undergraduate curriculum with 67% of academics refusing to allow dental students to select and bond orthodontic brackets [24]. However, it needs to be reiterated that GDPs continue to provide routine dental care to their patients who are receiving orthodontic treatment by specialist. Moreover, they may need to see patients experiencing problems with their orthodontic appliances, especially during emergency situations related to breakage of orthodontic appliances. Therefore, undergraduate dental students need to be provided with appropriate training to make basic adjustments to orthodontic appliances and also identify adverse effects of orthodontic appliances for timely management. Surveys show that most dentists do not feel prepared by their predoctoral education to apply orthodontic knowledge to clinical situations, with one survey reporting no significant improvement in clinical diagnostic skills from year one to year four [35]. Another study found that recent graduates did not recognize or refer a number of malocclusions in their general practice [38]. Foster-Thomas et al. found that there was a lack of confidence in undertaking orthodontic assessments in a cohort of newly qualified dentists who graduated from universities across the UK and overseas [39].

The need for achieving consistency in undergraduate orthodontic education was evidenced by several Delphi studies aiming to develop a consensus on orthodontic teaching in undergraduate programs [6]. A common message emanating from the available studies on undergraduate orthodontic education seems to be that teaching is focused on assessment and diagnosis of the orthodontic treatment needs of patients and developing a basic understanding of contemporary treatment options to facilitate patient referral. Undergraduate dental programs may find it challenging to train their students in providing orthodontic treatments apart from dealing with orthodontic emergencies [32]. Previous studies on the preparedness of dental graduates have also identified assessment and diagnosis of orthodontic treatment needs as the core skill required by new dental graduates [25,40].

It is acknowledged that some degree of variation in orthodontic teaching and assessments among individual institutions is inevitable. Nevertheless, there is merit in evaluating the alignment of LOs outlined by regulators, dental education bodies, and existing undergraduate orthodontic curricula used by dental education providers. In contrast to orthodontics, there appears to be greater consistency in undergraduate curricula for other major clinical dental disciplines, such as restorative dentistry, periodontics, endodontics, oral surgery etc. [26,41,42,43]. Relevant stakeholders need to critically evaluate the LOs that can be realistically achieved and focus on consolidating core skills rather than attempting to follow plans that are overly ambitious and unlikely to be feasible. In this regard, meaningful consultations are required amongst dental education providers, regulators, dental education associations such as ADEE and ADA, and specialist orthodontic associations such as the European and American orthodontic associations and similar organizations in individual countries. Last but not least, it is important to reiterate that dental students are the key stakeholders and must be represented appropriately in reviewing undergraduate curricula [44,45]. Such consultations may help achieve clarity and a broad-based consensus regarding the LOs and competencies in undergraduate orthodontic curricula.

The main limitation of this review was that it only explored published literature on undergraduate orthodontic education and did not attempt to dissect orthodontic curricula of individual dental institutions. Although the latter exercise may have provided additional information to answer the research questions in this study, given the fact that there are over a thousand dental schools worldwide, it was not feasible to explore individual curricula. It is also acknowledged that some studies included in this review were more than 20 years old and their findings may not accurately reflect the current education in respective institutions. Nevertheless, the review included some recent studies from Europe, the USA, and the Asia-Pacific region. The documents published by regulators and dental education associations in Europe, the USA, and Australia were also explored to identify the learning outcomes of orthodontic education in undergraduate dental curricula in order to articulate the common messages from key stakeholders in orthodontic education.

This study also highlights that there is a paucity of published research on orthodontic education in undergraduate curricula and underscores the need to explore the views of key stakeholders regarding the scope and structure of didactic teaching and clinical training in orthodontics; evaluate the validity and reliability of assessment methods; and identify appropriate strategies to enhance the learning experience of undergraduate students in orthodontics.

## 5. Conclusions

Limited published research is available on the topic and marked variations were observed both globally as well as regionally. There is a lack of consensus regarding the LOs, curriculum content, expected competencies, and assessment methods in undergraduate orthodontic education. Dental education providers, regulators, and professional bodies need to work closely to review existing undergraduate orthodontic curricula in order to align the LOs with teaching and assessments, thereby achieving consistency in the design and delivery of undergraduate orthodontic education.

## Data Availability

The data underlying this article will be shared on reasonable request to the corresponding author.
